# Targeting the Nutritional Value of Proteins From Legumes By-Products Through Mild Extraction Technologies

**DOI:** 10.3389/fnut.2021.695793

**Published:** 2021-07-19

**Authors:** Barbara Prandi, Chiara Zurlini, Cigognini Ilaria Maria, Sara Cutroneo, Martina Di Massimo, Marika Bondi, Andrea Brutti, Stefano Sforza, Tullia Tedeschi

**Affiliations:** ^1^Food and Drug Department, University of Parma, Parma, Italy; ^2^Stazione Sperimentale per l'Industria delle Conserve Alimentari, Parma, Italy; ^3^Conserve Italia Soc. Coop. Agricola, San Lazzaro di Savena, Italy

**Keywords:** legumes (fabaceae), agri-food by-products, proteins, extraction method, enzymatic hydrolysis

## Abstract

Legumes have been known for centuries for their good nutritional properties. Unfortunately, during processing, from 5 to 25% of this production is wasted, generating by-products that can still be a rich source of useful compounds, such as proteins, which can still be used in food and feed formulations. The choice of the extraction technique is important to preserve the nutritional value of proteins since drastic conditions of pH and/or temperature could damage them. In this work, two mild extraction techniques (direct assisted extraction—DAE and enzymatic assisted extraction—EAE) were applied for protein extraction from legume by-products obtained from agro-industrial processes. The quality of proteins was evaluated considering protein integrity [SDS-PAGE, degree of hydrolysis (DH), free amino acid content, racemization degree] and nutritional features [amino acid score (AAS), digestibility]. Direct assisted extraction is the technique that has best preserved protein integrity (1–5% DH and free amino acid content <1%), The digestibility of proteins extracted with EAE is higher (no protein bands detected in SDS-PAGE) than with the one of DAE extracts, making this technique particularly suitable for those food and feed formulation were a high digestibility of proteins is required.

## Introduction

Legumes (or pulses) belong to the *Fabaceae* family, and they have been known for centuries for their good nutritional properties. They have a high protein content and a good amino acid profile, with adequate levels of lysine; sulfurous amino acids and tryptophan are usually the limiting amino acids of this family (1). Among the negative aspects of legumes we find, however, the presence of some anti-nutritional factors, such as trypsin inhibitors and lectins, phytic acid, and tannins. These molecules are usually inactivated during the cooking process (2), but they must be considered when producing protein extracts that could be used in products not subjected to adequate heat treatment. In 2017 legumes had a market value worldwide of 44.9 billion U.S. dollars, with a production volume of 42.33 million metric tons [FAOSTAT (3), last accessed March 18, 2020]. The main legumes produced worldwide (in 2016) were beans, chickpeas, peas, and lentils. The quantity of legumes produced globally is therefore consistent, but unfortunately not all production is then translated into food. In fact, during food processing, from 5 to 25% of this production is wasted for several reasons: non-compliant beans (for density, size, or appearance), pods, leaves, or stems are discarded during the processing chain of fresh legumes (4). However, these by-products can still be a rich source of useful compounds, such as proteins, fibers, minerals, lipids, and phytochemicals (5). An option for the valorization of legume by-products is the extraction of valuable compounds, such as proteins. Protein isolates with high protein purity have already been produced from chickpeas, by using the protein rich residue obtained after the wet-milling of chickpeas (6). Different techniques have been developed and applied for the extraction of proteins from by-products of the agri-food sector. Protein extraction can be performed in dry-conditions (i.e., milling, sieving) or in wet conditions (i.e., chemical/biochemical/physical treatments) (7). Furthmore, enzyme assisted extraction was used to recover the protein fraction from soybean hulls, with protein and peptides recovery of around 60% (8). Subcritical water extraction was applied instead for protein extraction from raw and defatted soybean meal, with protein recovery of around 50% (9). As far as we know, most the applications have been proved on soybean residues and only a few of these techniques, and in particular dry ones, have been applied to legumes. The selection of the technique for protein extraction is very important for the efficiency of the process, and for the characteristics of the final product. The main objective of this work is to recover and valorize the protein fraction from legume by-products by maximizing its nutritional properties, therefore suitable for end use in food and feed. The target is thus to preserve the essential amino acids content, digestibility, and to minimize the presence of antinutritional compounds. Among the different extraction techniques used to produce protein concentrates, several are gaining attention as being particularly mild, therefore particularly suitable for preserving the protein quality: direct aqueous extraction (DAE) and enzyme-assisted aqueous extraction (EAE). Direct aqueous extraction is characterized by extraction with phosphate buffer followed by a separation with a decanter, without the use of organic solvents, high temperatures, or extreme pH, and has already been used in the past to extract proteins from legumes (pea, lentil, fava bean, chickpea, and bean) to produce thermoplastic biopolymeric materials (10). The protein rich supernatant is then acidified at the isoelectric point, and the protein rich precipitate is separated and concentrated. Enzyme-assisted aqueous extraction is based on the activity of selected enzymes, mainly of the class carbohydrase (which helps in matrix degradation increasing protein solubilization) or protease. In particular, the latter cut proteins into shorter peptides, which are more soluble and easier to extract in aqueous media. Thanks to the milder extraction conditions in terms of pH and temperatures, EAE can increase the extraction yield and provide extracts of better quality, however, EAE has some limitations which should be overcome, such as the cost of the enzyme (which could be quite expensive), the difficulties of fully hydrolyzing complex matrices (such as plant cell walls), and the possible modification of enzymatic activity in the scale up process (11).

In this work, for the first time, DAE and EAE (using different proteases) were compared for protein extraction from peas and chickpeas by-products. The composition, nutritional quality and the protein integrity of the above by-products was first determined, then DAE and EAE were applied to obtain several protein concentrates. These extracts were fully characterized in terms of protein integrity and of nutritional quality, evaluating protein and amino acid profile, degree of hydrolysis (DH), racemization degree, free amino acids content and digestibility, assessing their potential to further reuse in food and feed products.

## Materials and Methods

### Sampling of the Feedstocks

Chickpeas and peas feedstocks were provided by Conserves France (Saint Sylvestre sur Lot, France) and they were sampled both at their French and/or Italian production plants.

The pea residues were fresh, while the chickpea by-products were re-hydrated. For each kind of feedstock, different lots have been considered and examined. Each lot is different for variety or production day, to have the most significant representation of the feedstocks. Three different lots of fresh peas were sampled in three different days (14/06/2018, 06/06/2018, and 28/05/2018) and they correspond to three different varieties (ADOUR, XP0826, and SV794405). The lots have been collected at the exit rejection of the blanching process. Similarly, the re-hydrated chickpea residues consisted of three different lots, sampled in two different days (08/11/2018 and 20/11/2018), and they are all from the same variety (PASCIÁ, coming from Italy). The lots have been collected at the sorting, where an optical sorter divided the samples based on the color, moreover the samples were sorted based also on the dimensions.

### Direct Aqueous Extraction

The extraction was based on Stazione Sperimentale per l'Industria delle Conserve Alimentari (SSICA) patent (12), where neutral conditions have been identified as the best extraction conditions, with the advantage of a higher environmental and economic sustainability than alkaline conditions.

Laboratory scale extraction was performed starting from 100 g of each feedstock. In details, the starting by-products were washed with a vegetable washer machine to eliminate ground and dust residues, ground and homogenized by means of a blade mill in laboratory, while in pilot plant a colloidal mill has been utilized. No further pre-treatment for legumes byproducts was necessary, in fact, in all legume by-products examined samples, no inert materials (residues of leaves, pods, soil, small stones) were observed. After the homogenization of the starting feedstocks, the extraction was carried out with phosphate buffer. In particular, the by-products were treated with a neutral phosphate buffer (0.05 M Na_3_PO_4_ and 0.1 M NaCl, pH 7.2) in a 1:2 solid to liquid ratio. The treatment in phosphate buffer lasted at least 3 h, under stirring and at room temperature. Then, a separation step through decanter followed with the purpose to separate, on one hand, the liquid protein rich fraction, and, on the other hand, the solid fiber-rich fraction. In the first trials at laboratory scale, this separation step was carried out with a centrifuge. Instead, the proteins were isolated from the liquid protein-rich fraction thorough acidification at their isoelectric point (pH 4.5) through the addition of HCl 0.1 N, followed by a centrifugation step. The solid obtained from the centrifugation was finally lyophilized.

### Enzyme-Assisted Aqueous Extraction

The protein fractions of legumes (chickpeas and peas) were extracted by enzyme-assisted extraction using specific proteases (alcalase, trypsin, pepsin, papain, and combination of alcalase and papain) starting from 8 g of each feedstock. The feedstock was coarsely ground with a kitchen grinder and mixed with 40 ml of reaction media—phosphate buffer 10 mM (for alcalase, papain, trypsin, and mix), or hydrochloric acid 10 mM (pepsin). Then, the enzyme with an enzyme to substrate ratio of 1% (w/w for pepsin, papain, and trypsin, v/w for alcalase) was added. The extraction was carried out under constant stirring (water bath with magnetic stirrer) for 2 h at the following pH and temperatures: alcalase pH 6.5–8.5, T 60°C; trypsin pH 7–9, T 37°C; pepsin pH 2–4, T 37°C; papain pH 6–7, T 65°C; mix of alcalase and papain pH 6.5–7, T 62.5°C. As control, the extraction was carried out using the same conditions of time, pH, and temperature but without the enzyme. The protein supernatant was separated from the pellet by centrifugation (3,220 g for 20 min at room temperature) and lyophilized.

### Proximate Analysis

Dry residue, Protein, and Fat were determined according to the official methods for vegetable products (D.M. 3/2/89-G.U., Italian National Unification Body (13, 14).

The content of sucrose, D-glucose, and D-fructose was determined with a commercial kit by R-Biopharm, accordingly to the manufacturer instructions.

Regarding sugars, the sucrose, D-glucose, and D-fructose content is always determined on the basis of a UV method first published by Boehringer Mannheim GmbH, with a commercial kit from R-Biopharm. More in detail, the concentration of D-glucose is determined before and after the enzymatic hydrolysis of sucrose and the D-fructose is determined after the determination of D-glucose. As regards the determination of starch, the series of enzymatic reactions envisaged by the method involves the reduction of nicotinamide-adenine dinucleotide phosphate, the quantity of which is stoichiometric to the concentration of the components to be determined, found by the absorption of light at 340 nm. The sucrose content is calculated from the difference in D-glucose concentrations before and after the enzymatic inversion. All testing procedures were performed in duplicate in two technical replicates each. Results are expressed as mean-AVG (*n* = 2) ± *SD* in grams over 100 g (g/100 g).

Starch was determined with the same kit, after hydrolysis of starch to D-glucose at pH 4.6, in the presence of the enzyme amyloglucosidase.

### SDS-PAGE

The dry protein extracts were dissolved in 0.1 M HCl (50 mg in 10 ml) or in 75 mM HCl in 25% acetonitrile and 75% water (25 mg in 4 ml) depending on their solubility. Electrophoresis and identification of the protein bands were carried out as described in a previous work (15). Briefly, a sample volume corresponding to 30 μg of protein was dried under nitrogen flow and reconstituted with reducing sample buffer (sample buffer XT 4× and reducing agent XT 20×, suitably diluted with distilled water; Biorad, Hercules, CA, USA). Electrophoresis was performed at constant voltage (150 V) on Criterion XT Bis-Tris Gel at 12% (Biorad, Hercules, CA, USA) using the running buffer XT MES 20× (Biorad, Hercules, CA, USA) appropriately diluted with distilled water (duration about 45 min). The protein bands were stained with Coomassie Brilliant Blue R-250 (Biorad, Hercules, CA, USA) 1% w/V (dissolved in 50% distilled water, 40% methanol, and 10% acetic acid). Three to four destaining steps were performed with 50% distilled water, 40% methanol, and 10% acetic acid to achieve the desired contrast. The most intense bands in the feedstocks were cut from the gel and subjected to gel digestion after bleaching (washing steps with ammonium bicarbonate and acetonitrile), reduction (with dithiothreitol), and alkylation (with iodoacetamide), using trypsin as an enzyme. The peptides obtained from gel digestion were analyzed with μHPLC-LTQ-OrbiTRAP and the data were processed using the Peaks Studio software (Bioinformatic Solutions Inc., Waterloo, ON, Canada). The protein databases used were *Cicer arietinum* for chickpeas and *Pisum sativum* for peas.

### Total Amino Acid Analysis

Five hundred milligrams of feedstock (or 100 mg of dry protein extract) were weighed into L Pyrex glass tubes fitted with Teflon-lined screw caps. Six milliliters of 6 M HCl were added to each sample and mixed slowly. The tubes were flushed with nitrogen for 1 min to remove air. Hydrolysis was then carried out at 110°C for 23 h. After the tubes were cooled at room temperature, the internal standard (7.5 ml of nor-leucine 5 mM in deionized water) was added, and the mixtures were filtered through paper filter. The filtered solutions were collected into 250 ml volumetric flasks and brought up to volume with deionized water. Acid hydrolysis was used for the determination of all amino acids except tryptophan (Trp), cysteine (Cys), and methionine (Met). To determine the quantity of Cys and Met, they were oxidized with performic acid by incubating the samples overnight (in an ice bath) with 2 ml of freshly prepared performic acid. The next morning, 0.3 ml of hydrobromic acid (48%) was added to remove excess performic acid and the samples were dried under nitrogen flow. Then, acid hydrolysis was carried out as previously described. The hydrolyzed samples, as well as the amino acids standard mixture, were derivatized with AccQ Fluor reagent kit (Waters, Milford, MA, USA) following the manufacturer's instructions. The separation and detection of derivatized amino acids was achieved by RP-UPLC/ESI-MS using the Single Ion Recording acquisition mode. The analytical system is an Acquity UPLC coupled to a single quadrupole SQD detector (Waters, Milford, MA, USA), and the chromatographic column used is an Acquity BEH UPLC 300 A, 150× 2.1 mm with a C18 stationary phase (Waters, Milford, MA, USA). Details of the chromatographic and acquisition parameters are described in Buhler et al. (16).

Tryptophan was determined after alkaline hydrolysis as described by Caligiani et al. (17), using 5-methyltryptophan as an internal standard. Briefly, 300 mg of sample was added with 4 ml of 4 M sodium hydroxide and incubated for 4 h at 100°C. After alkaline hydrolysis, the samples were neutralized with 37% hydrochloric acid and filtered through 0.45 μm syringe filters. The filtered samples were added with the internal standard (150 μl of 0.7 mM 5-methyltryptophan) and the volume was made up to 10 ml with distilled water. Tryptophan and 5-methyltryptophan were determined by RP-UPLC/ESI-MS (Acquity UPLC coupled to a single quadrupole SQD detector, Waters, Milford, MA, USA) using an Acquity BEH UPLC 300 A, 150 × 2.1 mm column with a C18 stationary phase (Waters, Milford, MA, USA) and acquisition in single ion recording mode.

### Free Amino Acid Analysis

Ten microliters of sample (prepared as described for the SDS-PAGE analysis), as well as the amino acids standard mixture, were derivatized with the AccQ Fluor reagent kit (Waters, Milford, MA, USA) following the manufacturer's instructions. The separation and detection of derivatized amino acids were achieved by RP-UPLC/ESI-MS using a single quadrupole in Single Ion Recording acquisition mode. Details are described in Buhler et al. (16).

### Racemization Degree of Amino Acids

Forty milliliters of solution from the standard acid hydrolysis of each protein extract was dried by rotavapor. The dry sample was reconstituted with 2 ml of 2 M hydrochloric acid in 2-propanol and the reaction was carried out at 90°C for 1 h. Then, the samples were dried under nitrogen flow and reconstituted with 1 ml of dichloromethane and 0.5 ml of trifluoroacetic anhydride and incubated for 30 min at 50°C. The samples were dried again under nitrogen flow and reconstituted with 1 ml of dichloromethane just prior to analysis. The separation and detection of the derivatized amino acids was achieved by GC-MS using a Chirasyl-L-Val column. Details are reported in Anzani et al. (18).

### Hydrolysis Degree (*o*-phtaldialdehyde Analysis, OPA)

The dry protein extracts were dissolved in 0.1 M HCl (50 mg in 10 ml) or in 75 mM HCl in 25% acetonitrile and 75% water (25 mg in 4 ml) depending on their solubility. The DH, which is defined as the percentage of cleaved peptide bonds relative to the total number of peptide bonds, is calculated using the *o*-phthaldialdehyde (OPA) method, according to a previously published paper (19). Briefly, 20 μl of sample (or standard L-isoleucine solution) was mixed with 2.4 ml of OPA reagent (5 mM *o*-phthaldialdehyde, 5 mM N-acetylcysteine, 1% sodium dodecyl sulfate, 75 mM buffer borate in 10% methanol and 90% distilled water, pH 9.5). The absorbance was measured at 340 nm.

### Simulated Gastrointestinal Digestion

The dried protein fractions were subjected to simulated gastrointestinal digestion according to the INFOGEST method (20), starting from 100 mg of dried protein fraction (regardless of its protein content), and adapting the volumes of the digestive juices to maintain the same proportions. Briefly, 100 μl of simulated saliva (containing 75 U/ml of α-amylase) was added and the samples were incubated at 37°C with constant shaking for 5 min to simulate the oral phase. Then, 200 μl of simulated gastric juice (containing 2,000 U/ml of pepsin) was added and the sample was incubated under constant stirring at 37°C for 2 h to simulate the gastric phase. The samples were then added with 400 μl of simulated duodenal juice (containing 100 U/ml of pancreatin and 10 mM of bile) and incubated for 2 h at 37°C under constant stirring to simulate the duodenal phase. At the end of the digestion, the samples were heated to 90°C for 10 min to inactivate the enzymes thus stopping the digestion. The supernatant was recovered by centrifugation (13,000 g at 4°C for 10 min) and subjected to SDS-PAGE and to the determination of the DH.

### Statistical Analysis

Statistical analyzes (homogeneity of variance, *t*-test, one-way ANOVA—Duncan *post-hoc* test, pairwise comparison of types, independent-samples median test) were performed using SPSS Statistic version 26.0 (Statistical Package for Social Science, Chicago, IL, USA).

## Results and Discussion

### Legume by-Products Characterization

**Figure 1:**
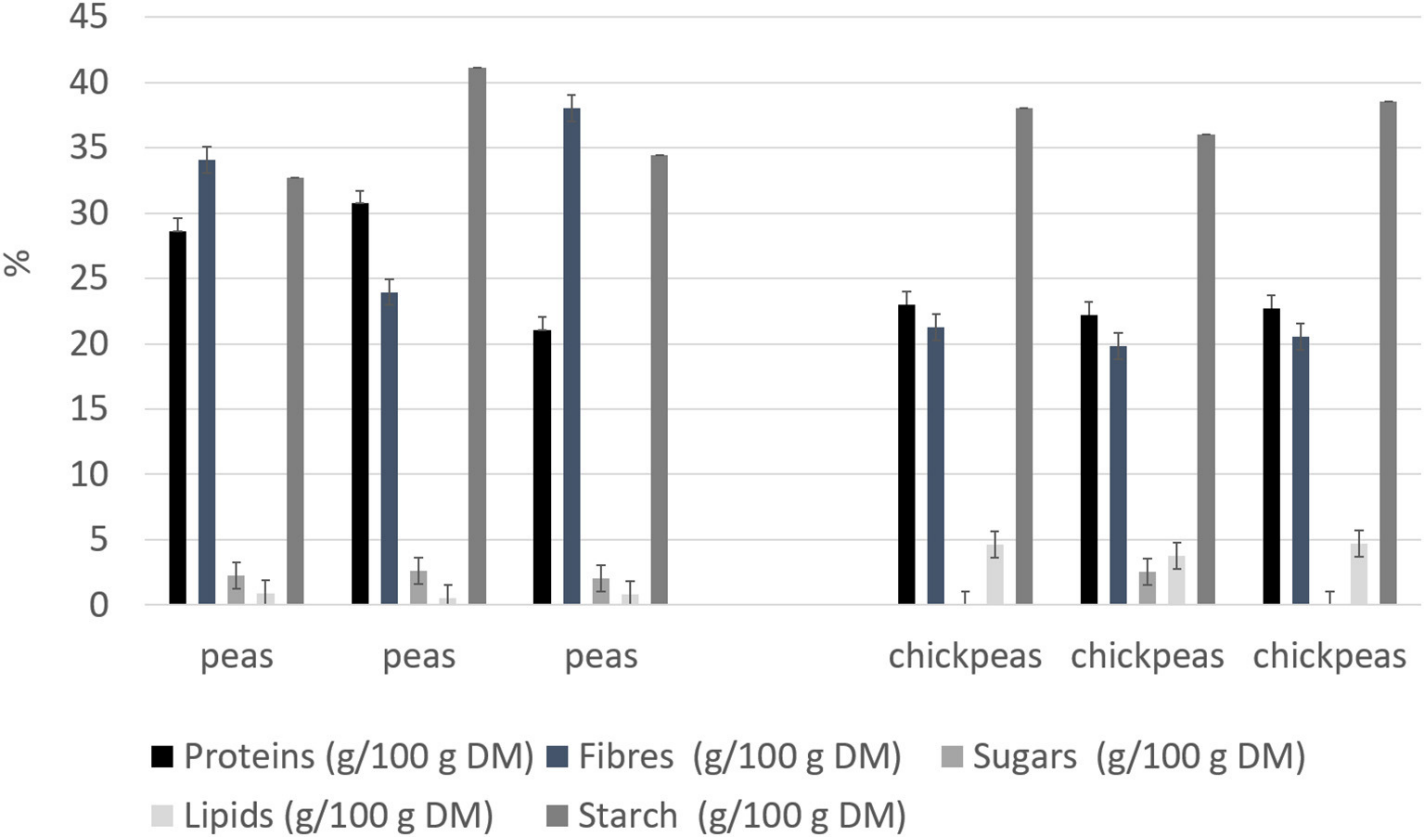
Nutritional composition for legume by-products (on dry weight). Results are reported for each of the three batches analyzed.

**Figure 2:**
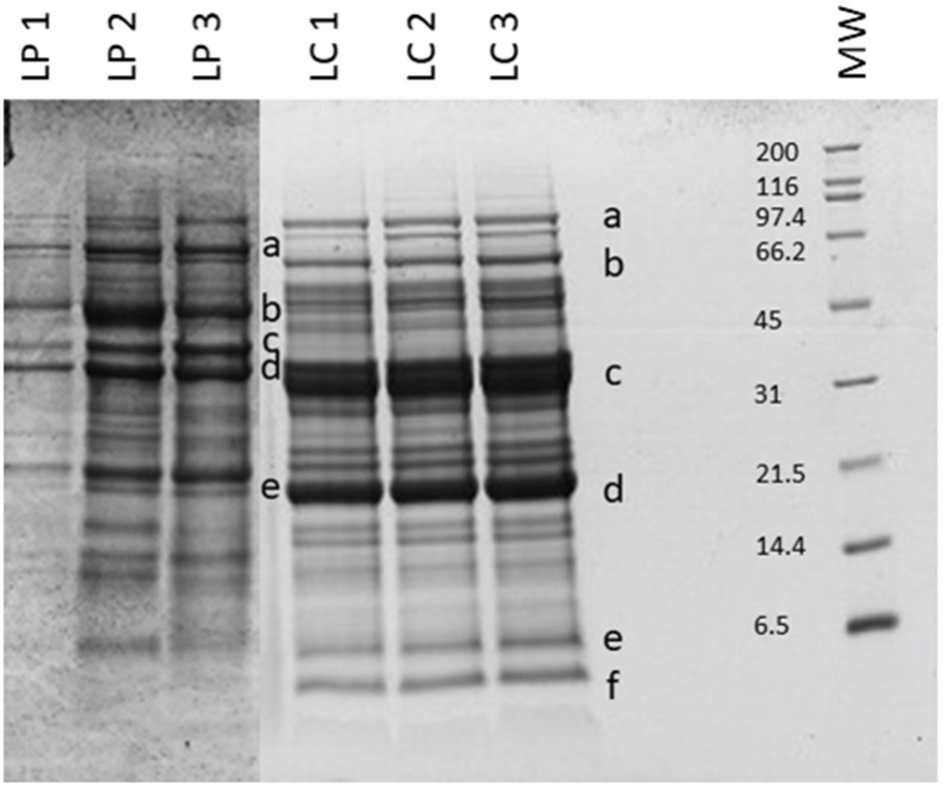
SDS-PAGE of the legume by-products. MW, molecular weight (in kDa). Protein identification (in gel digestion followed by LC-HRMS analysis): LP—a, convicilin; b, vicilin; c, legA class; d and e, 47k vicilin; LC—a, lipoxygenase; b and c, vicilin like; d, legumin J-like; e, legumin-like; f, 2S albumin-like. Results are reported for each of the three batches analyzed.

Beans and peas industrial processed by-products generally consist of non-conforming peas, skins, and plant parts. Especially pods are still present after the field harvest. The first part of the work was focused on the initial feedstocks (peas, and chickpeas) characterization (Figure 1). The knowledge of the feedstock composition is essential to verify the suitability of the by-product to produce high-quality proteins sources. No statistical differences were found among batches for the macronutrients tested (independent-samples median test, *p* < 0.05). The protein contents and, in general, also the other nutritional contents are in accordance with the nutritional tables from INRAN (Italian Institute of Food and Nutrition Sciences): this indicates that the composition of the by-products does not differ from the composition of the vegetable from which arise. As far as the protein content is concerned, all two byproducts showed a good amount, about 20–30% on dry matter basis. Analysis of the protein profile was performed by SDS-PAGE (Figure 2). By combining trypsin in gel-digestion of the bands and high-solution mass spectrometry, it was possible to identify the major storage protein classes, known in pea legumes (LP) and chickpea legumes (LC) such as vicilin and legumin. Moreover, LP and LC by-products showed DH below the 5% (Supplementary Figure 1A), indicating that no (or limited) proteolytic events occurred, in agreement with what observed in SDS-PAGE analysis Finally, protein integrity was also checked by analyzing the racemization degree of the amino acids. It is well known that amino acids racemization can occur in food (18) for different reasons: thermal stress (high temperature for long times), extreme pH (alkaline or acid conditions), microbial fermentation (certain D-amino acids are part of the bacterial cell wall). Alanine, aspartic acid, glutamic acid showed a very limited degree of racemization, together with some other amino acids. (Supplementary Figure 1B) Besides protein amount and integrity, also the amino acid composition, hence the nutritional value, is very important for determining the suitability of the proteins as food or feed constituents or supplements. Total amino acid composition was determined for all the samples (Supplementary Table 1), and the amino acid score (AAS) was calculated using the egg proteins as reference (21). The AAS can give an indication of the nutritional quality of the proteins, considering its composition in essential amino acids and the limiting amino acid as compared to the reference protein. The limiting amino acid found in LP was His and Met in LC. The nutritional value, calculated as AAS, of chickpea by-product proteins was 0.51, also consistent with literature data (22), and 0.37 for peas. Chickpea by-products showed good amounts of lysine and arginine, with the limiting amino acid being Met, according to values found for legumes (23). Pea by-products also had Met in very few amounts, as it is generally found in legumes (24).

### Protein Extraction From Peas and Chickpeas Feedstocks

Two mild techniques were applied for protein extraction from the selected feedstocks described above: DAE and enzyme assisted aqueous extraction (EAE) (using different commercial proteases) (details on the experimental procedures are reported in the Materials and Methods section).

The efficiency of each technique was calculated as the ratio between the quantity of extracted proteins and the total amount of proteins in the feedstock (in %). The extraction efficiency is an important parameter to determine if the process is economically sustainable. As shown in Table 1, the protein extraction yield is comparable between peas and chickpeas for EAE, while DAE has a higher (*p* < 0.05) extraction efficiency for chickpeas than peas. As far as DAE is concerned, very high efficiencies (up to 69%) could be achieved for chickpeas., Then, it was found that EAE allowed in general a two-fold increase in the protein extraction, with yield that was found from 10 to 22%, in the samples extracted without enzymes, to 25–48% in samples extracted using the enzymes as processing aids. The only exception was represented by the samples extracted with pepsin, probably due to the low pH used for the optimal activity of the enzyme, which is close to the isoelectric point of many pea and chickpea proteins (25). Thus, the use of proteases seems to affect the protein extraction yield, cutting the protein into shorter (and more soluble) peptides. Moreover, an effect of matrix degradation which promotes the solubilization and the extraction of the proteins was also probably present (7). Papain was the enzyme which showed the highest efficiency with extraction yields of 43% for LC and 58% for LP, respectively. The combined use of the two most performing enzymes papain and alcalase, anyway, did not show any synergistic effect (see results shown in Table 1).

**Table 1:** Protein extraction yields of direct aqueous extraction (DAE) and enzyme assisted extraction (EAE) on chickpeas and peas.

**Extraction condition**	**Protein extraction yield (%) chickpeas**	**Protein extraction yield (%) peas**
DAE	69 ± 16 (*n* = 4) (a)	10 ± 7 (*n* = 4) (f)
EAE (alcalase)	41 ± 1 (*n* = 2) (b, c, d, e)	42 ± 1 (*n* = 2) (b, c, d)
EAE (papain)	43 ± 1 (*n* = 2) (b, c, d)	58 ± 4 (*n* = 2) (a, b)
EAE (pepsin)	23 ± 1 (*n* = 2) (d, e, f)	12 ± 3 (*n* = 2) (f)
EAE (trypsin)	25 ± 8 (*n* = 2) (c, d, e, f)	30 ± 1 (*n* = 2) (c, d, e, f)
EAE (alcalase + papain)	34 ± 1 (*n* = 2) (b, c, d, e, f)	51 ± 1 (*n* = 2) (a, b, c)

*Equal letters correspond to values that are not significantly different (one-way ANOVA, Tukey's b post-hoc test; homogeneous variances—Levene test, p > 0.05). Comparison is made among all the combinations legume by-product × extraction condition*.

### Molecular Characterization of the Protein Extracts

#### Protein Content by Kjeldahl and Total Amino Acid Analysis

In addition to the mere extraction yield, it is also important to check the quality of the proteins extracted, depending on the final application, since the goal here is to produce high value proteins for food or feed. Table 2 shows the protein content (determined by Kjeldahl analysis) of the extracts obtained as described in section Protein Extraction From Peas and Chickpeas Feedstocks. These values are compared with the protein content based on the total amount of amino acids. The difference between the last two values also allowed to calculate the percentage of non-protein nitrogen on the total nitrogen. This last % is quite relevant in vegetal matrices, due to the presence of other natural non-protein compounds containing nitrogen (26). The data show that protein samples obtained with DAE technology from chickpeas had the highest protein content (*p* < 0.05, pairwise comparison of types, independent-samples median test), and the highest extraction yield (see Table 2): this implies that the method is selective in extracting mostly proteins with little impurities. On the other hand, EAE technology had higher yields of extraction for peas, and the proteins extracted with papain were the purest.

**Table 2:** Total nitrogen determination of the different extracts produced from chickpeas and peas feedstocks.

**Sample**	**Protein content (%DM) chickpeas^a^**	**Protein content (%DM) peas^a^**	**Protein content based on total amino acids value (% DM) chickpeas^b^**	**Protein content based on total amino acids value (% DM) peas^b^**	**Non-protein nitrogen on total nitrogen (%) chickpeas^c^**	**Non-protein nitrogen on total nitrogen (%) peas^c^**
DAE (lab scale)	74.1 ± 0.0^a^	65.2 ± 0.2^b^	58.0 ± 0.1	41.0 ± 0.3	22	35
EAE (alcalase)	37.1 ± 0.5^d^	37 ± 0.7^f^	28.6 ± 0.4	33.8 ± 0.2	26	11
EAE (papain)	46.5 ± 2.8^b^	66 ± 0.1^a^	35.5 ± 0.5	49.1 ± 0.3	27	26
EAE (pepsin)	35.8 ± 1.1^d^	45 ± 0.1^d^	23.5 ± 0.1	44.1 ± 0.1	37	2
EAE (trypsin)	35.4 ± 1.5^d^	47 ± 0.8^c^	25.4 ± 0.1	41.6 ± 0.2	27	13
EAE (alcalase + papain)	38.9 ± 0.6^c^	39 ± 1.1^e^	28.0 ± 0.3	31.0 ± 0.2	15	21

*Equal letters mean statistically equal values (pairwise comparison of types, independent-samples median test), The number of replicates was n = 2*.

a *Determined by Kjeldahl analysis*.

b *Determined by sum of total AA (with the exclusion of water)*.

c *Determined by subtracting the amount determined in ^b^ from the amount determine in ^a^ and expressing it in % on a*.

#### Protein Profile by SDS-PAGE

All the protein fractions obtained were then deeply analyzed for the assessment of the protein integrity. The protein profile of the extracts was first analyzed by SDS-PAGE (Supplementary Figure 2). Before loading onto the gel, protein samples were evaporated to dryness and reconstituted with the same protein buffer. For both peas and chickpeas extracts, we can observe a very rich protein profile in the DAE, fully consistent with that of the initial feedstock, meaning that the protein fraction is conserved during the extraction process, which uses very mild conditions, both in terms of pH and of temperature. On the opposite, no or few protein bands can be observed in the EAE samples, as expected, since the proteolytic enzymes only allow the extraction of the protein fraction in form of peptides or free amino acids. Residual protein bands remain in peas samples extracted with enzymes, whereas chickpeas proteins seemed to be totally hydrolyzed by all the enzymes. Therefore, DAE proteins are suitable for food or feed applications where the presence of whole proteins is required, for example to meet the desired techno-functional properties.

#### Protein Integrity by Evaluation of Degree of Hydrolysis and Amount of Free Amino Acids

Further analyses were performed to assess protein integrity. Protein integrity we mean the integrity of protein structure, in particular if this is affected after the application of the two different extraction protocols. This parameter can be evaluated by measuring the hydrolysis degree, the amount of free amino acids, the amount of D-amino acids residues (racemization degree).

The DH is an indicator for the cleavage of peptide bonds and the breakdown of the complex and structured proteins into smaller peptides and free amino acids. The amount of free amino acids is also reported for each sample (Table 3). Results are consistent with SDS-PAGE profile: DAE showed a low DH% and negligible (even if detectable) amount of free amino acids. The absence of proteolytic events confirms the good management of the legume by-products, and the mildness of the extraction process, which perfectly preserve the protein fraction. As expected, the free AA amount is higher in EAE (*p* < 0.05, pairwise comparison of types, independent-samples median test) than in DAE, because proteolytic activity releases free AAs. This can bring some improvements, such as a better digestibility, and could also favor a partial destruction of anti-nutritional factors, as confirmed by further analyses.

**Table 3:** Degree of hydrolysis (DH%) and amount of free amino acids (% w/w) in the dry protein extracts.

**Extraction condition**	**DH %^a^ peas**	**DH %^a^ chickpeas**	**Free amino acids (% DM) peas**	**Free amino acids (% DM) chickpeas**
DAE lab scale	1.52 ± 0.74^a^	5.3 ± 0.6^a^	0.05 ± 0.01^e^	0.07 ± 0.00^e^
EAE alcalase	42.9 ± 2.4^a^	12.6 ± 2.4^a^	4.3 ± 0.1^b^	6.3 ± 0.0^b^
EAE papain	12.4 ± 3.7^a^	11.0 ± 1.5^a^	5.4 ± 0.2^a^	7.8 ± 0.5^a^
EAE pepsin	32.5 ± 11.2^a^	14.8 ± 0.5^a^	2.5 ± 0.0^d^	3.5 ± 0.2^d^
EAE trypsin	23.5 ± 1.5^a^	13.7 ± 4.5^a^	5.5 ± 0.1^a^	4.9 ± 0.2^c^
EAE (alcalase + papain)	20.2 ± 1.3^a^	15.7 ± 4.0^a^	4.0 ± 0.0^c^	6.1 ± 0.3^b^

*Equal letters in the same column mean statistically equal values (pairwise comparison of type, independent samples median test, p < 0.05)*.

a *Calculated by OPA method. The procedure is described in Material and Methods section*.

Glutamic acid, arginine, and leucine were the most abundant free AAs found in chickpeas. Especially for Glu, this may have an influence on the taste features of the extract, since Glu is known to be a key factor in umami taste (27). The relative quantity of free AAs is generally in agreement with the total amino acid distribution, indicating that free AAs are released mostly according to their abundance, with only few exceptions probably due to protease specificities. Therefore, EAE extraction is more suitable for those food and feed applications were a high digestibility (and or a higher presence of free amino acids) is required. Moreover, the presence of free amino acids and short peptides will affect the taste and aroma of the final products, so a tailored enzyme extraction can optimize the final product characteristics, by modifying free amino acid and peptide patterns.

#### Protein Integrity by Evaluation of Degree of Racemization

A certain amount of D-Ala, and D-Asp were detected in all the protein extracts, both from DAE and EAE. However, with both the techniques, their amount was quite limited (always below 7.0% for DAE and below 5.5% for EAE) and fully comparable with the quantity of D-amino acids present in the initial feedstock (Supplementary Figure 3), indicating that no fermentative processes or harsh conditions were experienced by the samples during extraction. This data further confirms the suitability of DAE and EAE for the extraction of proteins with a high quality. These results are in agreement with pervious data reported in literature (28) on the determination of racemization degree of different food streams from agri-food sector.

The results demonstrate that both the techniques seem to be effective in extracting proteins from legumes feedstocks, with good extraction yields and negligible stress on protein fraction. The main difference between the two extraction methods is the form in which the proteins are extracted: DAE allows to obtain intact whole proteins, in their original composition, with high purity; instead, EAE method extracts the nitrogen fraction in form of a complex mixture of peptides and amino acids, with higher yields, but lower purity.

### Nutritional Value and Digestibility of the Protein Extracts

Protein extracts obtained from legume by products can be used as ingredients for new formulations. According to the potential applications of these extracts in food and in feed, an important feature is their nutritional value, determined by the total amino acid profile. Thus, the total amino acid profile was analyzed again, and compared to the profile before the extraction. The detailed amino acid composition can be found in (Supplementary Table 2).

In the pea samples, histidine, which was already present in small quantities in the starting feedstocks, was no longer detected in the EAE extracts. This was probably due to the increased temperature used during the protease hydrolysis, which could have been promoting the further oxidation of the already little histidine present.

Going more in details into the amino acid composition, some amino acids showed a significantly different amount between DAE and EAE, as determined by the *t*-test (*p* < 0.05). In fact, for chickpea extracts, 11 of 16 amino acids (calculated as relative % of each amino acid on the total) showed significant differences. Glycine, threonine, lysine, glutamic acid (sum of Glu and Gln), histidine, and tyrosine are significantly higher in EAE, while isoleucine, leucine, methionine, phenylalanine, and arginine are significantly higher in DAE. Analogously, in pea extracts 8 of 16 amino acids were significantly different between DAE and EAE. More specifically, lysine is higher in EAE, while serine, leucine, aspartic acid (sum of Asp and Asn), glutamic acid (sum of Glu and Gln), methionine, histidine, and arginine are higher in DAE. The different relative amino acid composition between the two extraction methods indicates that a different protein pattern is extracted, which is reflected in a different relative amino acid composition.

**Figure 3:**
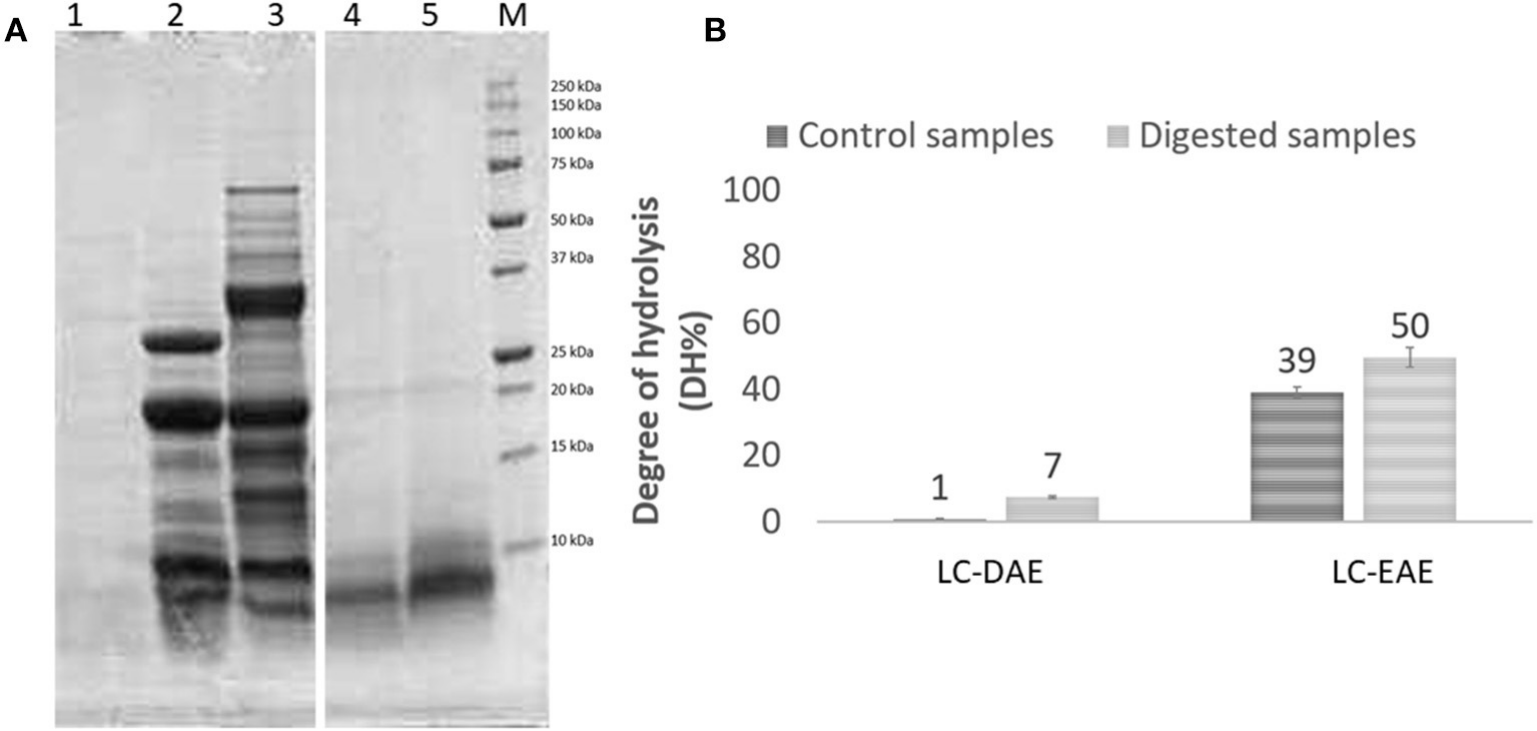
**(A)** SDS-PAGE profile and **(B)** degree of hydrolysis of chickpeas (LC) protein extracts, before (ctrl) and after (dig) digestion. 1, digestion blank; 2, LC-DAE dig; 3, LC-DAE ctrl; 4, LC-EAE (papain) dig; 5, LC-EAE (papain) ctrl. **(B)** determination of the degree of hydrolysis (DH%) by OPA method.

The AAS profile showed small variations in the essential amino acids content as compared to the same samples before extraction and for both the legumes the limiting AA is methionine, given the strong decrease above reported. The chickpea extracts, obtained with EAE with papain and alcalase, show an averagely significantly higher AAS (*t*-test, *p* < 0.05), compared to the initial feedstock (0.62 vs. 0.51). On the other hand, for pea samples, a small but not significant improvement in AAS is observed with DAE extraction (0.46 vs. 0.37). Linked to nutritional value, also the digestibility is an important parameter to determine the quality of a food or feed component. To evaluate the digestibility of these protein extracts, the harmonized INFOGEST *in vitro* digestion method (20) was applied. Among the various EAE extract, that obtained with papain was chosen for the test, given the better protein extraction yield of papain and the higher nutritional value if compared with the samples treated with other enzymes. After the *in vitro* digestion, samples were analyzed with SDS-PAGE to display the protein profile after digestion (Figure 3A). As expected, after *in vitro* digestion, the protein bands with MW > 30 kDa from DAE extracted samples, disappeared, as compared to the initial extracts, because they were cleaved by the digestive enzymes into shorter peptides and free amino acids. Some proteins with MW < 30 kDa are still present after digestion, indicating a partial digestibility of this protein fraction. The sample extracted with EAE, on the other hand, already proteolyzed, showed no meaningful changes in the high MW component after digestion.

To better quantify digestibility, the protein samples were also analyzed for their hydrolysis degree (DH%), and the results (Figure 3B) are fully consistent with those obtained from the SDS-PAGE. Prior to digestion, DAE proteins exhibit a very low DH, which means a high level of protein integrity. In the case of EAE, the papain used for the extraction has already proteolyzed the protein fraction, leading to a DH% of 39%. After the digestion, the hydrolysis degree increases for all the samples, due to the activity of the digestive enzymes. The values however remain quite low (not significantly higher after digestion, independent-samples median test*, p* < 0.05) for proteins obtained with DAE, with a DH% of 7% for chickpeas indicating a low digestibility. For EAE, the DH% increases further after digestion, reaching 50% (although not significant). These data seem to indicate that EAE extraction yields much more digestible protein fractions, since they are already more proteolyzed, at the end likely resulting in a better nutritional potential and better exploitation of the essential amino acids present. However, further studies on the digestibility of these extracts are required. The application of them as ingredients in new formulations and the digestion of the whole product should be investigated, to confirm the better digestibility of the protein fraction also in a formulated product.

## Conclusions

In the present study protein extraction from different typologies of legumes feedstock was approached for the first time by using two different mild extraction techniques, DAE and EAE.

The protein fraction of chickpeas and peas could be efficiently extracted both using DAE and EAE (especially using papain as enzyme). Direct aqueous extraction provided low protein extraction yields for peas, but the obtained protein extracts had a high degree of purity. Both the extracts showed to have a comparable protein quality, in terms of integrity and nutritional composition. The choice of the technique to better valorize proteins in the feedstocks is essentially based on the requirements of the final product. Direct aqueous extraction allows to obtain intact whole proteins, with a high purity degree, while EAE provides mixtures of peptides and amino acids with a high digestibility. According to the results here obtained, both the techniques could be easily implemented on an industrial scale.

The results here presented highlight the importance of performing a detailed chemical and molecular characterization of the protein fraction to select the best protein extraction technique and valorization route for agri-food residues. Moreover, the amino acid abundance in the different protein extracts can be very useful for the subsequent protein exploitation from biomass, as an alternative protein source for food applications, where protein extracts from legumes can compensate the lack of some amino acids in other ingredients (7, 29).

The increased interest in legume by-product/waste streams lies mainly in the possibility of recovering high-quality proteins, which are characterized by high levels of palatability and digestibility and could be further used as feed for all forms of livestock. At the same time the production of protein extracts with different features (i.e., protein hydrolisates), can be useful for the development of food ingredients with improve functional properties (i.e., digestibility, bioactivity).

## Data Availability Statement

The data presented in the study are deposited in the Uniprot repository, accession numbers (Q9M3X6, P13918, Q9T0P5, D3VND9, D3VND2, A0A1S2XBN2, A0A1S2Y087, A0A1S3E1A0, A0A1S2XVG1, A0A1S2XTK6, and A0A1S2XDF0).

## Author Contributions

SS: conceptualization, supervision, and writing—review and editing. MB: samples provider. SC, MD, AB, CM, and CZ: investigation and methodology. BP: conceptualization, investigation, methodology, and writing—original draft. TT: conceptualization, project administration, supervision, and writing—review and editing. All authors contributed to the article and approved the submitted version.

## Conflict of Interest

MB was employed by the company Conserve Italia Soc. Coop. Agricola. The remaining authors declare that the research was conducted in the absence of any commercial or financial relationships that could be construed as a potential conflict of interest.

## Supplementary Material

Supplementary Figure 1 **(A)** Degree of hydrolysis of legume by products determined by OPA method. **(B)** Percentage of D amino acids on the total content of each amino acids. Results are reported for each of the batches analyzed.

Supplementary Figure 2 SDS-PAGE of the protein extract obtained from peas **(A)** and chickpeas **(B)**, using direct aqueous extraction (DAE) or enzyme assisted extraction (EAE).

Supplementary Figure 3 Percentage of D amino acids on the total content of each amino acid. Results are reported for each protein extract obtained.

Supplementary Table 1 Determination of total amino acids content in the raw material.

Supplementary Table 2 Determination of total amino acids content in the protein extracts.

Supplementary Table 3 AAS calculated for all the samples object of this study.
