# Supplementary figures and images for: Targeting the Nutritional Value of Proteins From Legumes By-Products Through Mild Extraction Technologies
Source: Front Nutr. 2021 Jul 19;8:695793. doi: 10.3389/fnut.2021.695793 (PMC8326449; doi:10.3389/fnut.2021.695793)

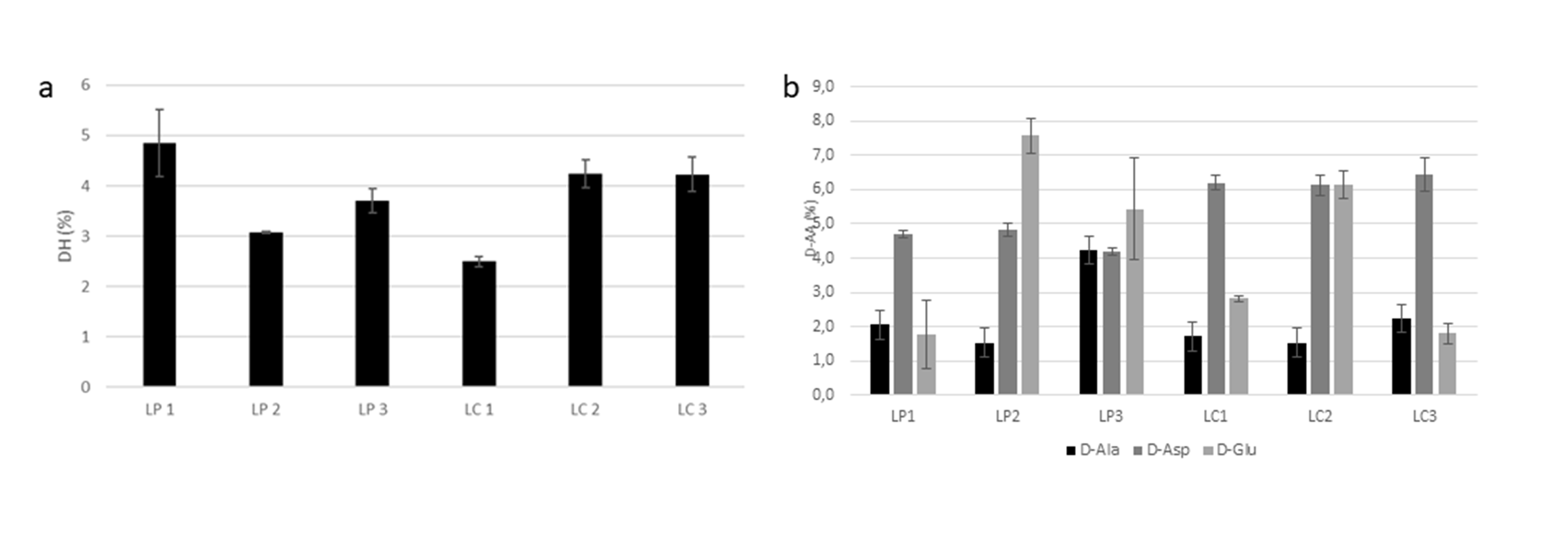

Supplement: Supplementary Figure 1 — (A) Degree of hydrolysis of legume by products determined by OPA method. (B) Percentage of D amino acids on the total content of each amino acids. Results are reported for each of the batches analyzed. [file Image_1.TIF]

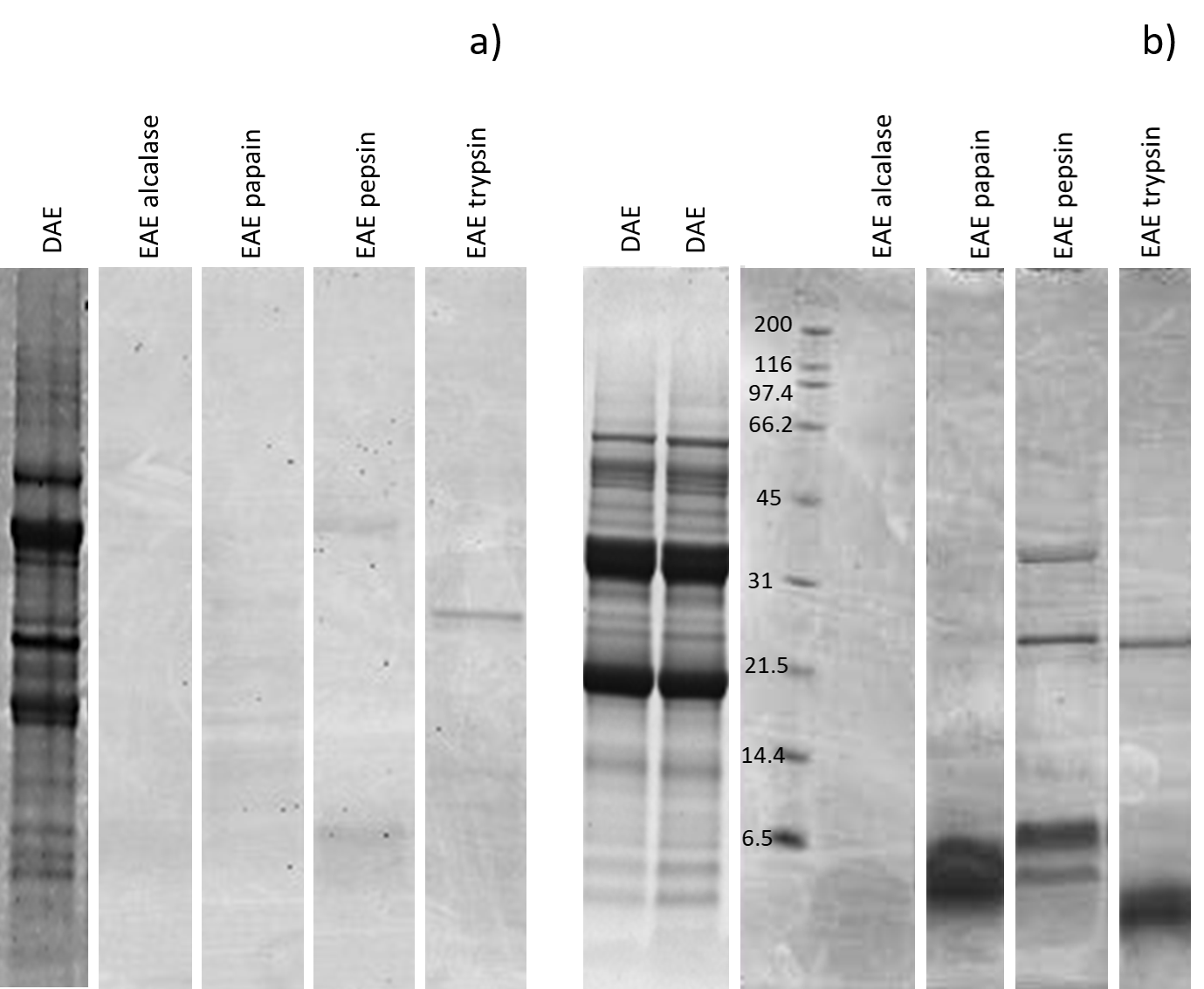

Supplement: Supplementary Figure 2 — SDS-PAGE of the protein extract obtained from peas (A) and chickpeas (B), using direct aqueous extraction (DAE) or enzyme assisted extraction (EAE). [file Image_2.TIF]

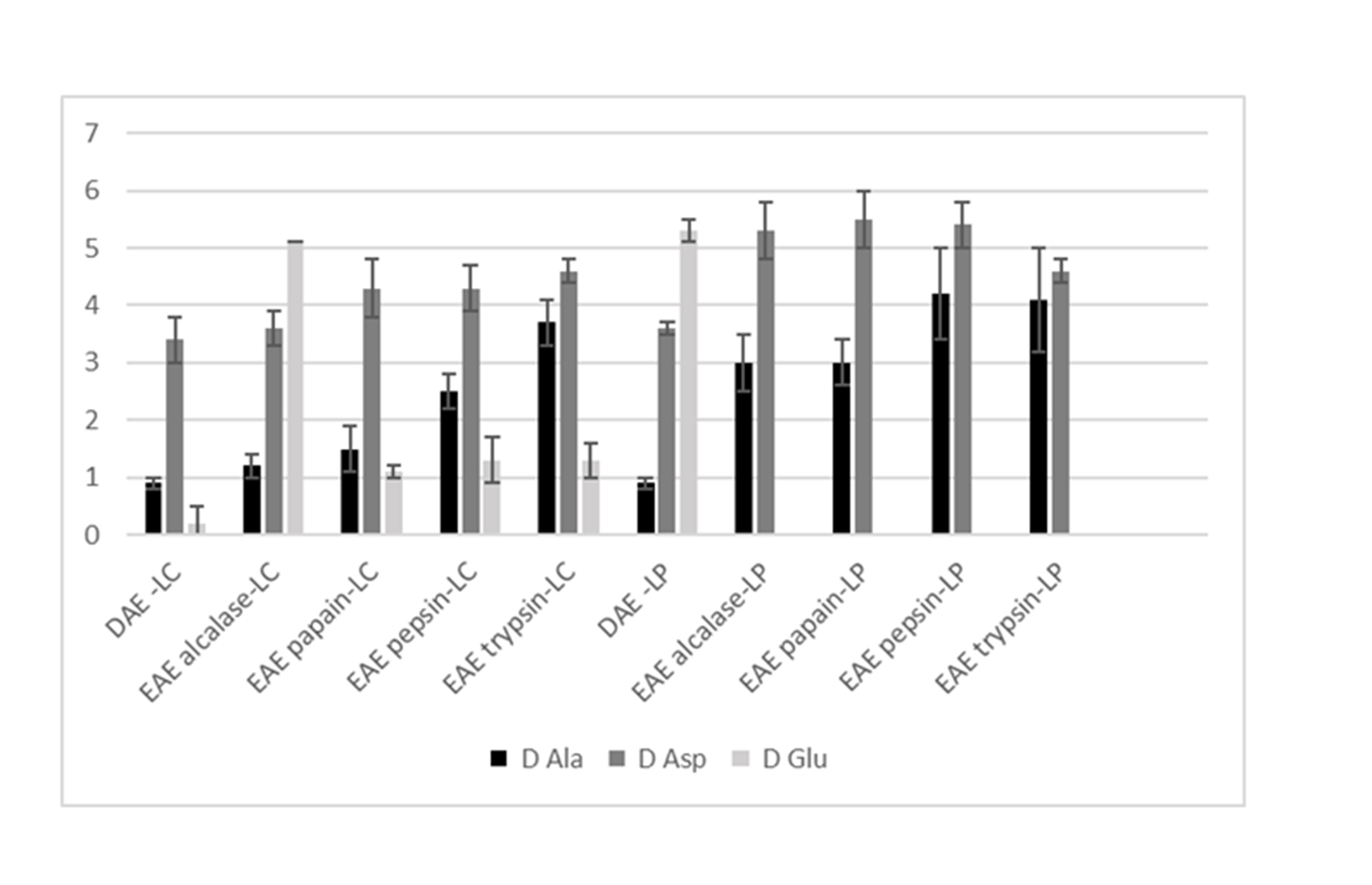

Supplement: Supplementary Figure 3 — Percentage of D amino acids on the total content of each amino acid. Results are reported for each protein extract obtained. [file Image_3.TIF]
