# Supplementary material for: Targeting the Nutritional Value of Proteins From Legumes By-Products Through Mild Extraction Technologies
Source: Front Nutr. 2021 Jul 19;8:695793. doi: 10.3389/fnut.2021.695793 (PMC8326449; doi:10.3389/fnut.2021.695793)
Supplement: Supplementary Table 1 — Determination of total amino acids content in the raw material. [file Table_1.pdf]

Results are the mean of two replicates (n=2).

AMINO ACIDS (% w/w, fresh weight)

Sample	Code	Gly	Ala	Ser	Pro	Val	Thr	Ile	Leu	Asp	Lys	Glu	His	Phe	Arg
	LP 1	0,21	0,23	0,25	0,20	0,27	0,19	0,22	0,41	0,53	0,29	0,73	0,05	0,26	0,38
PEAS	LP 2	0,22	0,25	0,29	0,24	0,30	0,22	0,26	0,47	0,63	0,35	0,86	0,05	0,30	0,46
	LP 3	0,23	0,27	0,28	0,23	0,29	0,22	0,24	0,45	0,60	0,34	0,87	0,05	0,29	0,53
	LC 1	0,31	0,34	0,44	0,36	0,42	0,32	0,38	0,67	0,92	0,56	1,34	0,23	0,52	0,68
CHICKPEAS	LC 2	0,33	0,35	0,47	0,39	0,47	0,36	0,42	0,75	1,03	0,64	1,50	0,25	0,58	0,88
	LC 3	0,30	0,36	0,44	0,38	0,42	0,31	0,39	0,68	0,89	0,59	1,43	0,23	0,50	0,80

AMINO ACIDS (% w/w, dry weight)

Code	Gly	Ala	Ser	Pro	Val	Thr	Ile	Leu	Asp	Lys	Glu	His	Phe	Arg
LP 1	0,94	1,06	1,13	0,92	1,24	0,88	1,00	1,87	2,41	1,32	3,32	0,21	1,18	1,71
LP 2	1,17	1,30	1,53	1,23	1,58	1,13	1,35	2,44	3,26	1,84	4,49	0,25	1,55	2,42
LP 3	0,94	1,11	1,15	0,95	1,19	0,88	0,99	1,81	2,43	1,37	3,53	0,20	1,16	2,15
LC 1	0,69	0,76	0,98	0,80	0,92	0,71	0,83	1,48	2,04	1,25	2,96	0,50	1,14	1,51
LC 2	0,66	0,69	0,93	0,77	0,92	0,70	0,83	1,48	2,04	1,26	2,97	0,50	1,16	1,75
LC 3	0,65	0,76	0,95	0,81	0,91	0,67	0,83	1,45	1,90	1,26	3,06	0,49	1,06	1,70

Tyr	Cys	Met	Trp	AA sum
0,14	0,09	0,08	0,26	4,8
0,16	0,09	0,07	0,18	5,4
0,14	0,23	0,13	0,23	5,6
0,20	0,15	0,13	0,54	8,5
0,23	0,15	0,15	0,42	9,4
0,21	0,14	0,14	0,34	8,5

Tyr	Cys	Met	Trp	AA sum
0,64	0,43	0,37	1,18	21,8
0,86	0,49	0,38	0,94	28,2
0,58	0,94	0,51	0,93	22,8
0,45	0,32	0,30	1,19	18,8
0,45	0,30	0,30	0,83	18,6
0,44	0,29	0,29	0,73	18,3
