# Supplementary material for: Targeting the Nutritional Value of Proteins From Legumes By-Products Through Mild Extraction Technologies
Source: Front Nutr. 2021 Jul 19;8:695793. doi: 10.3389/fnut.2021.695793 (PMC8326449; doi:10.3389/fnut.2021.695793)
Supplement: Supplementary Table 2 — Determination of total amino acids content in the protein extracts. [file Table_2.pdf]

AMINO ACIDS (% w/w, dry weight)

Results are mean values of two replicates (n=2).

% AA (w/w dry extract)	*Gly*	*Ala*	*Ser*	*Pro*	*Val*	*Thr*	*Ile*	*Leu*	*Asp*	*Lys*	*Glu*	*His*	*Phe*	*Arg*	*Tyr*	*Cys*	*Met*	*sum AA*
EFAE LC lab scale	2,7	3,1	3,8	3,1	3,3	2,3	3,2	5,6	8,3	2,6	12,0	1,6	4,6	7,4	2,0	0,7	0,8	67,2
EFAE LP lab scale	2,3	2,2	2,7	2,2	2,7	2,0	2,4	4,3	5,4	2,4	8,0	1,2	2,5	4,0	1,8	0,7	0,7	47,4
EAE LC alcalase	1,4	1,5	1,9	1,4	1,5	1,3	1,3	2,5	4,1	1,5	6,1	0,9	1,9	2,6	1,1	0,7	0,6	32,0
EAE LC papain	1,9	1,8	2,2	1,7	1,9	1,7	1,7	2,9	5,0	1,9	7,7	1,1	2,2	3,1	1,4	1,3	0,9	40,2
EAE LC pepsin	1,6	1,3	1,4	1,3	1,2	1,3	1,1	1,7	3,0	1,4	4,4	0,9	1,3	2,3	1,0	0,5	0,3	26,2
EAE LC trypsin	1,2	1,3	1,6	1,3	1,4	1,2	1,3	2,3	3,6	1,6	5,7	1,0	1,7	2,3	1,0	0,7	0,4	29,6
EAE LC mix (alcalase + papain)	1,4	1,5	1,8	1,4	1,5	1,3	1,4	2,4	4,0	1,6	6,2	0,9	1,8	2,8	1,1	1,0	0,8	33,0
EAE LP alcalase	2,1	1,7	2,1	1,6	2,2	1,7	2,1	3,3	4,3	3,1	5,7	0,0	2,3	2,6	1,3	0,4	0,5	37,0
EAE LP papain	3,2	2,5	3,3	2,5	3,2	2,5	2,8	4,7	5,7	4,2	8,6	0,0	3,9	4,5	2,6	1,0	0,6	55,9
EAE LP pepsin	4,4	3,0	3,1	3,3	2,6	2,4	2,2	3,5	5,6	3,6	7,7	0,0	2,6	3,6	1,7	0,6	0,4	50,2
EAE LP trypsin	2,0	2,2	2,8	2,1	2,5	1,9	2,4	4,0	5,3	4,4	7,9	0,0	2,7	3,2	1,7	0,4	0,5	46,0
EAE LP mix (alcalase + papain)	1,9	1,7	2,2	1,7	2,2	1,8	2,0	3,5	3,6	2,7	5,3	0,0	2,8	2,5	1,4	0,5	0,4	36,3
