# Supplementary material for: Targeting the Nutritional Value of Proteins From Legumes By-Products Through Mild Extraction Technologies
Source: Front Nutr. 2021 Jul 19;8:695793. doi: 10.3389/fnut.2021.695793 (PMC8326449; doi:10.3389/fnut.2021.695793)
Supplement: Supplementary Table 3 — AAS calculated for all the samples object of this study. [file Table_3.pdf]

Table 3S. AAS of all the samples object of these studies. In bold the limiting amino acid.

	LP 1	LP 2	LP 3	LC 1	LC 2	LC 3
His	**0.39**	**0.37**	**0.36**	1.11	1.12	1.11
Ile	0.70	0.72	0.66	0,67	0.68	0.69
Leu	0.98	0.98	0.90	0.90	0.91	0.90
Lys	1.15	1.23	1.13	1,25	1.29	1.30
Met	0.54	0.44	0.71	**0.51**	**0.51**	**0.52**
Phe	0.94	0.96	0.89	1.05	1.08	1.01
Thr	1.02	1.01	0.97	0.95	0.96	0.92
Val	0.77	0.76	0.71	0.66	0.67	0.67

	DAE LP	EAE LP alcalase	EAE LP papain	EAE LP pepsin	EAE LP trypsin	EAE LP mix
His	1.04	<LOQ	<LOQ	<LOQ	<LOQ	<LOQ
Ile	0.74	0.84	0.76	0.64	0.76	0.83
Leu	1.02	1.00	0.94	0.77	0.96	1.08
Lys	0.96	1.57	1.41	1.33	1.78	1.41
Met	**0.46**	**0.42**	**0.33**	**0.26**	**0.36**	**0.37**
Phe	0.91	1.06	1.21	0.88	1.02	1.31
Thr	1.06	1.13	1.12	1.19	1.04	1.20
Val	0.75	0.79	0.76	0.68	0.72	0.82

	DAE LC	EAE LC alcalase	EAE LC papain	EAE LC pepsin	EAE LC trypsin	EAE LC mix
His	0.98	1.10	1.11	1.34	1.34	1.12
Ile	0.71	**0.62**	0.63	0.63	0.66	0.63
Leu	0.93	0.86	0.82	0.74	0.86	0.82
Lys	0.73	0.86	0.86	1.03	0.98	0.93
Met	**0,40**	0.63	0.72	**0.41**	**0.42**	0.78
Phe	1.18	0.99	0.93	0.88	1.01	0.93
Thr	0.85	1.02	1.03	1.26	1.02	1.01
Val	0.65	0.62	**0.63**	0.62	0.62	**0.62**
